# Ethyl Acetate Fraction of *Helianthus tuberosus* L. Induces Anti-Diabetic, and Wound-Healing Activities in Insulin-Resistant Human Liver Cancer and Mouse Fibroblast Cells

**DOI:** 10.3390/antiox10010099

**Published:** 2021-01-12

**Authors:** Arokia Vijaya Anand Mariadoss, SeonJu Park, Kandasamy Saravanakumar, Anbazhagan Sathiyaseelan, Myeong-Hyeon Wang

**Affiliations:** 1 Department of Bio-Health Convergence, Kangwon National University, Chuncheon 200-701, Korea or drmavanand@kangwon.ac.kr (A.V.A.M.); or saravana732@kangwon.ac.kr (K.S.); or sathiyaseelan@kangwon.ac.kr (A.S.); 2 Chuncheon Center, Korea Basic Science Institute (KBSI), Chuncheon 24341, Korea; sjp19@kbsi.re.kr

**Keywords:** *Helianthus tuberosus*, antioxidant, anti-diabetic, insulin, wound healing

## Abstract

Traditional, complementary, and integrative medicine are globally accepted alternative methods for the treatment of diabetes mellitus (DM). However, the mechanism of anti-diabetic effects of *Helianthus tuberosus* L. remains unproven. In the present study, antioxidant and anti-diabetic activity of the tubers of *H. tuberosus* were studied in detail. Methanolic extracts of *H. tuberosus* tubers were subjected to solvent fractionation method by increasing the polarity of the solvent using *n*-hexane, and ethyl acetate. The obtained methanol extracts and its fractions were subjected to free radical scavenging activity (DPPH and ABTS assay) and in vitro enzyme (α-amylase and α-glucosidase) inhibition assay. Moreover, glucose uptake in insulin-resistant HepG2 cell line was analyzed. The preliminary phytochemical analysis confirmed the presence of phenolic and flavonoid compounds in the active fraction. The radical scavenging and in vitro diabetic related enzyme inhibitory activities were found to be dose dependent. The maximum ABTS^+^ and DPPH scavenging activity was documented in ethyl acetate fraction of the *H. tuberosus* followed by methanol extract, hexane fraction, and methanol fraction. We also found that *H. tuberosus* showed a less toxicity in mouse fibroblast cells and enhance the glucose uptake in insulin-resistant HepG2 cells. Besides, the ethyl acetate fraction of the *H. tuberosus* analyzed by UPLC-QTOF-MS-MS and GC/MS revealed the presence of phenolic compounds such as neochlorogenic acid, chlorogenic acid, caffeic acid, 5-*O*-(4-coumaroyl)-quinic acid, feruloylquinic acid, caffeoylquinic acid, isoxazolidine, salicylic acid β-D-glucoside, dicaffeoylquinic acid isomers, salvianolic acid derivative isomers, and 1,4 dicaffeoylquinic acid etc. Among the identified phytochemicals, six were chosen for molecular docking study to explore their its inhibitory interactions with α-amylase and α-glucosidase. Taken together, the findings of the present study suggested that phytocompounds of EAF were responsible for the significant in vitro antioxidant, wound-healing, and anti-diabetic activities.

## 1. Introduction

Diabetes mellitus (DM) is one of the common serious carbohydrate metabolic disorders reported globally and it is a gateway for several human diseases [1]. According to the WHO report, around 422 million people are suffering from DM. In last three decades, the incidence of DM has been drastically increased in Republic of Korea and it is estimated to be more than 150% in the years 2000 to 2035. [2]. Aging, obesity, and food habits are the common risk factors for DM. There is no available marketed food free from chemicals and these food items can cause several kinds of illness including DM [3]. Although DM cannot be completely cured, the regulation of healthy diet, and exercise would help escape from the severe impacts of the disease. It is best to maintain the diet by consuming food rich in protein and fiber, as a starchy diet increases blood sugar levels. Vegetables, fruits, whole grains, skimmed milk, dairy products, poultry, meat, and fish are the ideal foods for the management of diabetes [4].

Even though people are taking much care on their health, the epidemiology of DM is gradually increasing day by day. There are several hypoglycemic drugs to treat DM, which include synthetic, semi-synthetic, and chemical formulations such us metformin, sulphonylureas, pramilintide, thiazolidiones, etc., [5]. Globally, diabetes drug market is valued of 57000 million USD in 2018 and it is expected to reach 87000 million USD by 2025. However, these syntactic drugs cause severe side effects. A number of studies has documented the anti-diabetic mechanism of medicinal plants. There are several ancient traditional medicine systems, including Korean folk medicine, Chinese medicine, Japanese kampo medicine, Indian Ayurveda system, African complementary and alternative medicine, practiced for a long while to treat diabetes [6]. Particularly, there are numerous traditional medicinal plants reported to have hypoglycemic properties such as *Vinca rosea, Momordica charantia, Azadirachta indica, Trigonella foenum, Ocimum sanctum,* and *Allium sativum* [7,8,9]. Also, several research articles have described the isolation of functional molecules from natural resources to cure the DM. Using the search term of “functional food and DM” in PubMed dated on 28 December 2020 showed ~882 research articles. Intrigued by this, we intended to search and validate anti-diabetic compounds from functional food sources. *Helianthus tuberosus* L., commonly known as Jerusalem artichoke, is a traditional medicinal plant belonging to the family of sunflower plant [10]. Its therapeutic potential has been well documented in traditional, complementary, and alternative medicine for more than a century. In the mid-19th century, people used to consume it as an alternative to potato. In the past, *H. tuberosus* tuber was one of the important ingredients in Korean soup [11]. It is also one of the unique plants that contain all the essential amino acids, which are the necessary nutrients for humans. Also, the higher amount of polysaccharides, phenolic, and flavonoid compounds might be responsible for its medicinal property [12]. The leaves of *H. tuberosus* show anti-spasmodic, anti-inflammatory, anti-pyretic, anti-fungal, and analgesic activity. The tubers of *H. tuberosus* have shown spermatogenic, diuretic, cholagogue, and aperient activities and have been used in the treatment of rheumatism and diabetes [13,14,15]. Unauthorized folk medicine practitioners have reported that the plant material might have potential to ameliorate insulin sensitivity through decreasing the fasting glucose level in diabetic patients. Considering all these facts, the present study aims at the detailed scientific validation of antioxidant, wound-healing, and anti-diabetic effects of active organic fraction from *H. tuberosus*.

## 2. Materials and Methods

### 2.1. Chemicals and Cell Lines

The plant material of *H. tuberosus* was procured from Buan Dongjin farm, and the material was authenticated by Prof. MH Wang, Kangwon National University. Cell culture media, accessories, chemicals, and other reagents were procured from sigma Aldrich, Korea. The mouse fibroblast cell line (NIH3T3) and the hepatoma cell line (HepG2) were procured from Korea cell line bank, Korea.

### 2.2. Preparation of Plant Extract and their Active Fractions

A modified experimental procedure proposed by Teke et al. (2011) was employed for the extraction and active fractionation of *H. tuberosus* [16]. Briefly, the tubers part of the plant sample was allowed to shade-dry and ground to a coarse powder. Approximately 200 g of this powder was dissolved in 1000 mL of methanol and kept for 48 h and the extract was filtered. To the remaining mass, 250 mL of fresh methanol was added, allowed to stand further for 24 h, and the extract was filtered. Then the extracts were pooled together, and the excess solvent was removed by a rotary vacuum evaporator at a reduced pressure to get a concentrated extract. This concentrated extract (13.97 g) was used for further studies. To this, a portion of 10 mg extract pre-dissolved in 20 mL of methanol and 10 mL distilled water were added and then partitioned with 50 mL of *n*-hexane using a rotatory vacuum evaporator. The upper organic layer (n-hexane) was collected, and this step was repeated for two times. The semi-liquid concentrated fractions were collected and marked as an active fraction of hexane. The lower aqueous layer (aqueous methanol) was concentrated and mixed with 50 mL of ethyl acetate to collect the active fraction of ethyl acetate as mentioned above. The remaining lower layer (aqueous methanol fractions) was collected, labelled with respective fractions, and preserved for further use.

### 2.3. Estimation of Total Phenol and Flavonoid Content

According to the method described by Saeed et al. (2012), the total phenol and flavonoid content in the organic fractions of *H. tuberosus* (Ht-EAF: *H. tuberosus*-ethyl acetate fraction, Ht-HF: *H. tuberosus*-hexane fraction, Ht-MF: *H. tuberosus*-methanol fraction) and its methanolic extracts (HT-ME) were analyzed [17]. Briefly, 100 µL of sample (extracts/active fractions) was dissolved in 200 µL of Folin-Ciocalteu’s reagent (Sigma Aldrich, St. Louis, MO, USA) and subjected to gentle shaking. To this, 200 µL of 2% Na_2_CO_3_ was added, mixed thoroughly, and the mixture was kept in dark conditions for 30 min at room temperature. The absorbance of the mixture was recorded at 760 nm using a UV-visible spectrophotometer and the total phenolic content was determined using standard gallic acid curves. To estimate the total flavonoid content, 50 µL of sample (extracts/active fractions) was mixed with 200 µL of distilled water, 150 µL of 95% ethanol, 10 µL of AlCl_3_, and 10 µL of CH_3_COOK. Then, the mixture was kept for 45 min at room temperature and the absorbance was recorded at 415 nm against blank (without sample). Total flavonoid content was expressed in quercetin equivalents.

### 2.4. Determination of In Vitro Antioxidant Activity

Antioxidant activity of the *H. tuberosus* extract/active fraction was screened by ABTS^+^ assay, as described by Arnao et al. (2001) with a minor modification [18]. An ABTS tablet (A9941, Sigma Aldrich, St. Louis, MO, USA) containing 10 mg of ABTS^+^ was dissolved in 2.45 mM of potassium persulphate and methanol to make the stock solution. After 12 h of incubation period, the absorbance of the solution was recorded using a UV-vis spectrophotometer at 734 nm. Fresh working solution of ABTS^+^ was prepared by dissolving 250 µL of stock solution in 4.5 mL of ethanol. About 100 µL of sample (Ht-EAF, Ht-HF, Ht-MF, and HT-ME) was allowed to react with 100 µL ABTS^+^ in dark for 10–15 min and the absorbance was recorded at 734 nm. Similarly, DPPH^●^ radical scavenging activity was measured by the method described elsewhere. Briefly, 100 µL of *H. tuberosus* extract/active fraction was treated with 0.1 mM of DPPH^●^ (100 µL) solution, and the mixture was incubated for 10 min. The absorbance was recorded at 517 nm. Antioxidant activity in terms of % ABTS^+^ and DPPH^●^ radical scavenging activity were calculated by standard methods described earlier.

### 2.5. α-Amylase and α-Glucosidase Inhibition Assay

Anti-diabetic potential of the organic fractions of *H. tuberosus* (Ht-EAF, Ht-HF, Ht-MF) and its methanolic extract (HT-ME) was examined by a standard method of α-amylase and α-glucosidase inhibition assay. In brief, the reaction mixture consists of 50 µL of sample, 200 mM of phosphate buffer (pH 6.8), α-amylase as enzyme (Sigma Aldrich, St. Louis, MO, USA), starch as substrate, and DNS as a coloring agent. The reaction mixture was incubated for 15 min at 80 °C and its inhibition activity was spectrophotometrically read at 540 nm [19]. The method of Sathiyaseelan et al. (2020) was used to measure the inhibition activity of α-glucosidase [20]. Briefly, 25 µL of α-glucosidase was mixed with 50 µL of sample and incubated for 15 min. Then, 25 µL of the substrate solution (*p*-nitrophenyl-α-D-glucopyranoside) was added to the mixture. After the 15 min incubation period, 100 µL of saturated sodium carbonate (Na_2_CO_3_) solution was added to stop the reaction. All the steps in the protocol were carried out at room temperature and the inhibition activity was measured at 405 nm by spectroscopy. Acarbose was used as the positive control. The incubation activities were calculated using the following formula and IC_50_ values were measured using Graphpad Prism.

Inhibitory activity = [(A_c_ − A_t_)/A_c_] × 100 (1)

where, A_c_ is the absorbance of the control and A_t_ is the absorbance of test sample.

### 2.6. Biocompatible Nature of Ethyl Acetate Fraction of H. tuberosus

The mouse fibroblast cell line (NIH3T3) was used to evaluate the biocompatible nature of Ht-EAF. Briefly, NIH3T3 cells (1 × 10^5^) were cultured into 96-well plate and allowed for overnight incubation. Then the cells were washed with 100 µL of ice-cold PBS (Corning, NY, USA). Then, 10 µL of different concentration (2.5−50 µg/mL) of different fractions (hexane, ethyl acetate, and methanol) of *H. tuberosus* were dissolved in RPMI media (Sigma Aldrich, St. Louis, MO, USA) and placed in culture plates. Subsequently, the plates were incubated in a humidified 5% CO_2_ chamber. Then, 10 µL of WST solution (CellomaxTM, Mediflab, Seoul, Korea) was added to each well and incubated for a further 4 h to assess the cytotoxicity. The biocompatible nature of the test material was recorded (OD at 450 nm) using a multi-functional microplate reader. Additionally, the apoptotic-inducing nature (AO/EtBr staining) and loss of mitochondrial membrane potential (Rhodamine 123 staining) of the samples were screened by standard fluorescent staining method [21]. Photographs were captured using a fluorescent microscope (Olympus CKX53, Tokyo, Japan).

### 2.7. In Vitro Anti-Diabetic Nature of Ethyl Acetate Fraction of H. tuberosus

To ascertain the anti-diabetic nature of the Ht-EAF, cell cytotoxicity and glucose uptake assay were studied with HepG2 cells. For cytotoxicity studies, HepG2 cells (5 × 10^6^) were grown in high glucose DMEM media supplemented with 10% of fetal bovine serum and 1% antibiotic solution. Then, the culture flask was kept in a humidified 5% CO_2_ chamber and allowed to reach 80% confluence. Then, the cells were treated with varying concentrations of Ht-EAF to determine the cytotoxicity [22]. The method of Chen et al. (2019) was adopted for glucose uptake assay [23]. Briefly, HepG2 cells (1 × 10^5^) were fixed in a DMEM medium supplemented with 10% fetal bovine serum and 1% antibiotic solution and incubated for 12 h. Then, 5 × 10^7^ mol/L of human insulin (Sigma Aldrich, Gillingham, Kent, UK) containing fresh DMEM medium was added to the flask and kept for 48 h to create insulin resistance (IR) cells [24]. Later the IR-HepG2 cells were cultured in a 96-well plate and treated with varying concentrations of HT-EAF (5–50 µg/mL) and allowed to stand for 12 h. Non-resistance HepG2 cells were kept as the positive control in the experimental setup. After 12 h of incubation, the cells were collected, and the glucose content was measured by DNS assay. Besides, IR HepG2 cells were sub-cultured and used to assess the oxidative stress, mitochondrial membrane potential, and nuclear damage by fluorescent staining method [25].

### 2.8. In Vitro Wound Scratch Assay

Wound-healing potential of Ht-EAF was screened using NIH3T3 cells according to the method described by Mariadoss et al., 2020 [26]. Briefly, 2 × 10^5^ cells were seeded in a 12-well plate and kept in a 5% CO_2_ incubator at 37 °C to grow for full confluence. Afterwards, the cells were washed with ice-cold PBS solution and exposed to fresh medium. The monolayer of the cells was horizontally scratched with a 100-µL pipette tip to make an artificial wound and the cell debris was removed by washing with PBS. Then, the cells were treated with different concentrations (25, 50, and 100 µg/mL) of Ht-EAF and diluted with serum-free media of DMEM. The cells without treatment were considered as control cells. The cell migration assay was documented using a phase contrast microscope (20×) at different time intervals (0 h-initial, 12 h, and 24 h-mid and 48 h-final). The photograph was analyzed by ImageJ software and the wound closure exposure was determined by the following formula.

Wound closure = [(Measurement at 0 h-Measurement at 48 h)/Measurement at 0 h] × 100(2)

### 2.9. Anti-Bacterial Activity

The antibacterial activity of Ht-EAF was screened by agar well diffusion method [27]. The bacterial cultures such us *Staphylococcus aureus*, *Bacillus subtilis*, *Klebsiella pneumoniae*, and *Escherichia coli* were used to support the wound-healing activity. The bacterial samples were uniformly placed onto the surface of the MHA plate. Then, 20 µL of Ht-EAF sample (mg/mL) was carefully added to each well. The plates were incubated in upright position for 24 h at 37 °C. After incubation, the zone of inhibition was recorded using a ruler. In addition, the Live/Dead^TM^ BacLight^TM^ bacterial viability assay (Invitrogen, Waltham, MA, USA) was performed to strengthen the results obtained from previously discussed methods. The assay was performed following manufacturer’s instructions. The microphotograph was documented by phase contrast microscope using a green filter (Olympus CKX53, Tokyo, Japan).

### 2.10. UPLC-QTOF-MS/MS Analysis

Chromatographic separation and the detection of mass spectroscopic analysis of ultra-performance liquid chromatography-quadrupole/time of fight mass spectrometry (UPLC-QTOF-MS/MS) (WATERS XEVO GS-XS QTOF analyzer) was utilized for the identification of an active compound profile. About 2 μL of Ht-EAF sample was injected into the sample holder and the separation was carried out by using an Acquity UPLC BEH C18 column (50 × 2.1 mm, 1.7 μm) (Waters Co., USA). The flow rate of the sample was controlled at 0.3 mL/min and the column temperature was maintained at 30 °C. The column was evaluated by using 0.1% of formic acid in water (A) and 0.1% of formic acid in acetonitrile (B) and the separation was done by 10% A to 10% B gradient elution within 10 min. The phytochemicals were identified with the help of literature support acquired for Medicine, PubMed, Chemspider, Web of science and UNIFI 1.8 software.

### 2.11. GC-MS Analysis

Based on the antioxidant and antidiabetic assays, the ethyl acetate fraction of *H. tuberosus* (Ht-EAF) was used for the GC-MS analysis (Agilent 7890A, 5975C integrated with a Saturn 2000 mass spectroscopy detector, temperature limits: 60–325 °C, ionization voltage: 70 eV, columns: Agilent J&W GC columns (DB-5MS, 30 m × 0.25 mm with 0.25 µM thickness)). An aliquot of 1 µL of the sample was injected into the GC/MS apparatus. The operation conditions were: column oven program 60 °C (isothermal for 1 min) to 230 °C (isothermal for 3 min) at 3 °C/min and 250 °C for injection, detector temperature 300 °C. Helium was used as the carrier gas with a flow rate of 1.2 mL min^−1^. The W8N05ST.L mass spectral library was used to identify the phytochemical constituents.

### 2.12. Molecular Docking Study

The molecular docking study of selected phytocompounds (neochlorogenic acid, caffeic acid, cryptochlorogenic acid, caffeoylquinic acid, 3-(4-methylpiperidin-1-yl)propan-1-amine and feruloylquinic acid) against anti-diabetic target enzymes of α-amylase and α-glucosidase was performed by ArgusLab 4.01 and the docking outputs were visualized by using BIOVIA discovery studio visualizer V20. Docking process has several steps, which includes ligand and protein processing, conversion, optimization, and energy minimalization, etc. The structures of all the tested phytocompounds were retrieved from NIH PubChem (https://pubchem.ncbi.nlm.nih.gov/) and their energy minimalization was done by USCF chimera (Ver 1.14). The target proteins of α-amylase (IOSE) and α-glucosidase (3A4A) were retrieved from RCSB Protein Data Bank (PDB), and the non-protein parts (water molecules, other ligands, etc.,) were removed. The molecular docking of processed α-amylase/α-glucosidase with energy minimalized selected phytocompounds was analyzed by ArgusLab 4.01 programme. The size of the Grid box was fixed into maximum with default space. The results were visualized by using BIOVIA discovery studio visualizer V20 software.

### 2.13. Statistical Analysis

All the experiments were carried out in triplicate. GraphPad Prism software (Version 5.01) was used for statistical and graphical representation of the data. The data were expressed as mean ± SD. The statistical difference among the experimental groups was measured by using one-way ANOVA followed by Duncan’s analysis. *p* value of <0.05 was considered statically significant.

## 3. Results and Discussion

In the past five decades, researchers all over the global have been working toward the complete eradication and management of DM, as the increasing incidence rate would increase the mortality rate [18]. Among several therapies involving synthetic drugs, insulin therapy has received a great attention as it stabilizes the blood glucose level. However, repeated, and prolonged dosage of this drug causes severe adverse effects including skin rash, stomach upset, dizziness, shivering, nausea, and kidney complication. In this regard, plant extracts and their active compounds have been considered an attractive source of anti-diabetic drug to stabilize the blood glucose level without any harmful effects. In the recent past, the isolation and validation of anti-diabetic drugs from natural resources have gained attention. Usually, the therapeutic index of bioactive compounds from the plant sources are obtained in the form of terpenoids, saponins, tannins, alkaloids, glycosides, carotenoids, alkaloids, flavonoids, and phenolics [28]. Based on the above, solvent fractionation method was employed to collect phytochemical-enriched fractions of *n*-hexane, ethyl acetate, and methanol. Preliminary screening of phytochemicals and quantification of total phenolic content (TPC) and total flavonoid content (TFC) were done with *H. tuberosus* extracts following a standard spectroscopic method. Besides, the GS-MS analysis documented that several phytocompounds are present in the extracts and active fraction of the *H. tuberosus*, which are responsible for its antioxidant and anti-diabetic activity. In addition, α-amylase and α-glycosidase assays were carried out to document the anti-diabetic index of the *H. tuberosus* extracts and active fraction. Further, in silico docking studies were performed to validate the molecular interaction with the identified phytochemicals.

### 3.1. Phytochemical Quantification

The total phenolic content in the active fractions and the methanolic extract of *H. tuberosus* was determined by the standard Folin-Ciocalteu method and the results are collected in Table 1. Based on standard curve of gallic acid equivalent (GAE) [R^2^ = 0.9326], the total phenolic content was found to be 69.55 ± 0.36 mg GAE/g for Ht-EAF, 43.07 ± 0.05 mg GAE/g for Ht-ME, 17.11 ± 1.05 mg GAE/g for Ht-HF, and 10.29 ± 0.82 mg GAE/g for Ht-MF. The Ht-EAF has highest amount of phenolic content than the other fractions. The total flavonoid content was calculated against the equivalent of quercetin [R^2^ = 0.952]. The total flavonoid content varied from 6.27 ± 1.09 to 21.03 ± 4.36 mg of quercetin equivalent (QE)/g dried weight. The Ht-EAF has the highest amount of flavonoid content than the other fractions. It was found to be 21.03 ± 0.97 mg QE/g for Ht-EAF, 19.85 ± 0.21 mg QE/g for Ht-ME, 11.48 ± 0.11 mg QE/g for Ht-HF, and 6.27 ± 1.09 mg QE/g for Ht-MF. Taken together, the findings of the phytochemical quantifications in *H. tuberosus* revealed that phenols and flavonoids are abundantly present in the fractions that are responsible for its therapeutic potentials [29,30,31]. Moreover, the total flavonoid content was significantly lower than the total phenolic content. These results are in accordance with an earlier study reported by Nizioł-Łukaszewska et al. [32]. Therefore, the antioxidant and antidiabetic activities were screened by chemical and cellular assays and the possible mechanisms are discussed below.

### 3.2. Radical Scavenging Potential

**Figure 1:**
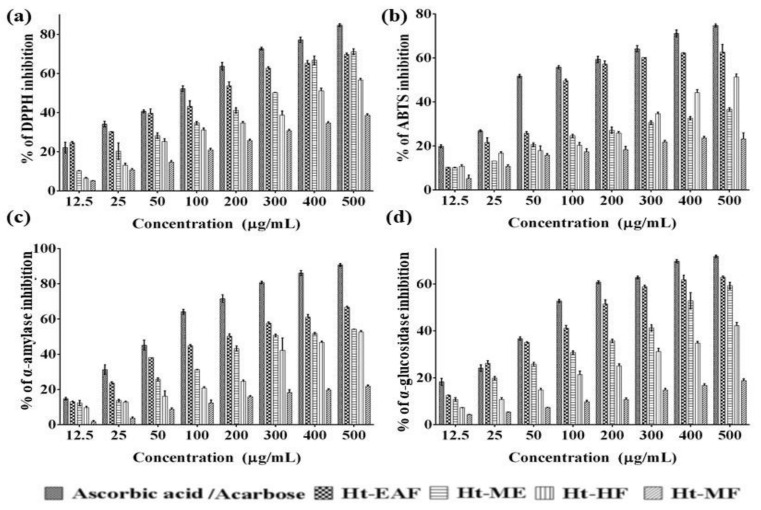
(**a**) % of DPPH (**b**) ABTS radical scavenging (**c**) α-amylase and (**d**) α-glucosidase inhibition activity of different fractions and methanolic extracts of *H. tuberosus*. The values are expressed as mean ± standard deviation of three independent experiments.

Phenolic-based phytoconstituents have diverse biological activities including anti-microbial, anti-inflammatory, anti-atherosclerotic, and anti-carcinogenic activity. These activities might be directly associated with the antioxidant properties of the medicinal plants. There are several in vitro assay models available to screen the radical scavenging properties of the plant extracts and their active metabolites. Among them, DPPH^●^ and ABTS^+^ are widely accepted and practiced by many researchers. The DPPH radical scavenging potentials of various fractions are depicted in Figure 1a. The results revealed that all the active fractions from *H. tuberosus* showed a concentration-based radical scavenging activity. The IC_50_ values of DPPH^●^ radical-scavenging activity of Ht-EAF, Ht-ME, Ht-HF, and Ht-MF was found to be 161.55 ± 0.98 μg/mL, 286.32± 1.21 μg/mL, 391.13 ± 4.52 μg/mL, and 756.23± 0.13μg/mL, respectively, while that of the standard ascorbic acid was 75.83 ± 0.61 μg/mL. Among the tested samples, the ethyl acetate fraction exhibited the strongest DPPH^●^ radical scavenging activity. The ABTS^+^ scavenging activities of *H. tuberosus* fractions are presented in Figure 1b. The IC_50_ value of ABTS^+^ radical scavenging activity of Ht-EAF, Ht-ME, Ht-HF, and Ht-MF was found to be 104.45 ± 3.01 μg/mL, 492.29 ± 1.87μg/mL, and 948.90 ± 2.09 μg/mL (<1000 μg/mL), while that of the standard ascorbic acid was 48.84 ± 2.04 μg/mL. The maximum ABTS^+^ scavenging activity was documented in ethyl acetate fraction of the *H. tuberosus* followed by methanol extract, hexane fraction, and methanol fractions (Figure 1b). The results were compared with the antioxidant activity of standard ascorbic acid. These findings are in line with the earlier studies, wherein concentration-dependent DPPH^●^ radical scavenging activity was recorded. The noted antioxidant activity is correlated to the phenolic profile of *H. tuberosus*. Concentration-dependent antioxidant and free radical scavenging activities have been reported in many phenolic-rich phytocompounds [33]. Overall, it could be suggested that the phenolic-based phytocompounds present in the active fraction of the *H. tuberosus* reduced the oxidative-stress-mediated complications in diabetic conditions.

### 3.3. In Vitro Anti-Diabetic Efficacy

There is an increasing demand to establish a natural inhibitor of anti-diabetic-targeting molecules formulated in the form of functional food, capsule, pill, etc. Due to less side effects and low cost, the plant-based anti-diabetic molecules have great ability in treating DM patients [34]. Among the many target molecules of antidiabetic drugs, α-glycosidase is an ultimate target for the therapeutic management of DM. It is one of the key enzymes that catalyze the final stages of digestion process in carbohydrate metabolism. The dysfunction of this enzyme accelerates the rise in post-prandial glucose level in DM [35]. Also, the α-amylase-inhibiting activity is explored in this study. The available literature evidently shows that inhibition of α-amylase activity lowers hyperglycemia. The three fractions have a significant inhibitory activity against α-glycosidase, which ranged from 1.73 to 77.74% (Figure 1c).

The IC_50_ values of Ht-EAF, Ht-HF, Ht-MF, Ht-ME, and acarbose against α-glycosidase were found to be 187.04 ± 0.42, 377.88 ± 1.63, <1000 and 723.58 ± 0.15 μg/mL, respectively. Besides, the α-amylase inhibiting activity ranged from 1.7 to 90.79%. The IC_50_ value of Ht-EAF, Ht-HF, Ht-MF, Ht-ME, and acarbose against α-glycosidase were found to be 102.53 ± 1.39, 294.39 ± 0.65, 456.92 ± 2.07, <1000 and 64.62 ± 0.59 μg/mL, respectively (Figure 1d). Among the three different fractions, ethyl acetate has more pronounced α-glycosidase- and α-amylase-inhibiting activity. From these findings, the active fraction of *H. tuberosus* was found to inhibit irreversibly α-glycosidase and α-amylase. In line with previous findings, it is suggested that the phytochemicals present in the extracts or phytochemical-rich fractions slowdown the breakdown rate of polysaccharides, reduce the blood glucose level, and consequently suppress the post-prandial glucose level in blood [36]. These preliminary findings are consistent with the previously documented data from Jdir et al., who documented the similar trends of results [37]. The results could be utilized for future work to determine the anti-diabetic compound from *H. tuberosus*.

### 3.4. Biocompatibility Nature of H. tuberosus

**Figure 2:**
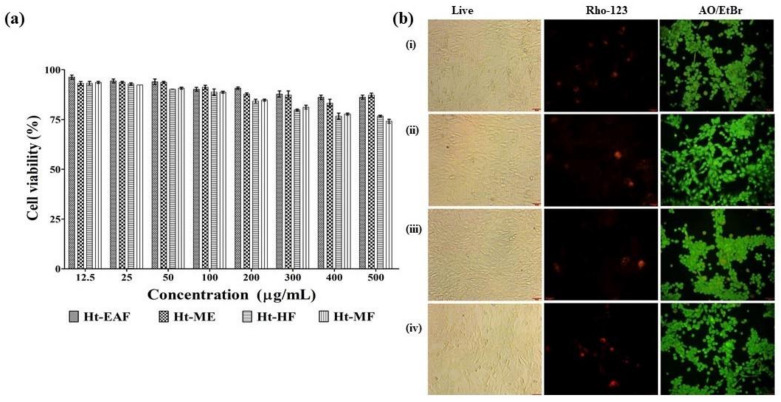
(**a**) % of cell viability of different fraction and methanolic extracts of *H. tuberosus*. (**b**) Biocompatibility of HT-EAF was screened by light microscopic, rhodamine 123, and dual staining. (i) CK, (ii) 12.50 µg/mL of Ht-EAF (iii) 200 µg/mL of Ht-EAF (iv) 500 µg/mL of Ht-EAF. The values are expressed as mean ± standard deviation of three independent experiments.

Morphological changes of the ethyl acetate fraction of *H. tuberosus* were evaluated by light microscopic analysis, nuclear damage was assessed by PI staining, and the apoptotic inducing potential was assessed by AO/EtBr staining method. First, the biocompatible effects of different fraction of *H. tuberosus* were studied in noncancerous cell line of NIH-3T3 (mouse fibroblast cells). The cytotoxicity nature assay was performed by WST assay. The results of the cytotoxic activity of all the fractions showed >76% cell viability (Figure 2a). The findings of the phase contrast microscopic images also confirm that there are no noticeable cytotoxic features (Figure 2b).

Cell integrity damages were not observed even at high concentration of ethyl acetate fraction of the *H. tuberosus*-treated cells. Similarly, the active fractions of this plant material do not cause any changes in the mitochondrial membrane as confirmed by Rhodamine 123 staining. Besides, the dual staining analysis revealed the absence of apoptotic bodies (Figure 2b).

### 3.5. Anti-Diabetic Effects of H. tuberosus

**Figure 3:**
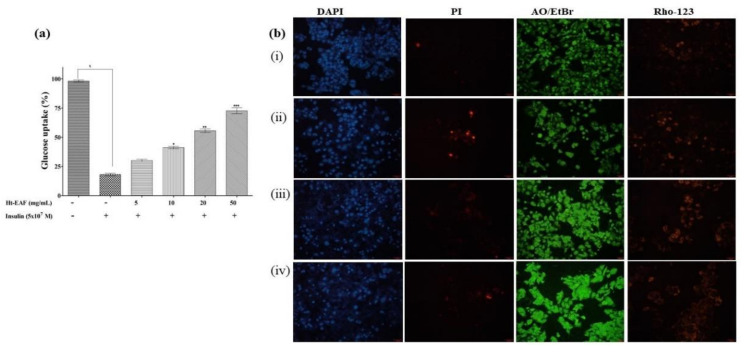
(**a**) Ht-EAF enhance the glucose uptake in insulin-resistant HepG2 cells. (**b**) Morphological changes of HT-EAF treated IR-HepG2 cells was screened by DAPI, PI, AO/EtBr, and rhodamine 123 and dual staining.(i) CK, (ii) 12.50 µg/mL of Ht-EAF (iii) 200 µg/mL of Ht-EAF (iv) 50 µg/mL of Ht-EAF. Data were expressed as mean ± standard deviation of three independent experiments. * *p* < 0.001 represent the statistically significant from the untreated HepG2 cells; * *p* < 0.05, ** *p* < 0.01, and *** *p* < 0.001 represents the significant differences from the 5 x 10^7^ M insulin-treated control group.

HepG2 cells are an ideal cell culture model for the study of hepatic glucose uptake and insulin resistance pathway. Insulin resistance in liver cells is mainly caused by the impaired glycogen production, which is the main contribution to hyperglycemia [38]. Based on this, insulin-resistant HepG2 cells were used for this study to explore the anti-diabetic effects of Ht-EAF as shown in Figure 3a. The addition of 5 × 10^7^ mol/L of insulin incubation of HepG2 cells showed the decreased level of glucose uptake. Moreover, the treatment with Ht-EAF showed concentration-based glucose uptake in IR-HepG2 cells and it confirmed that the active fraction treatment has the ability to prevent the impairment of glucose uptake. As expected, the 50 µg/mL of Ht-EAF increased the glucose uptake nearly 75%. Our results also clearly confirm that 5 × 10^7^ mol/L of insulin treatment significantly induce the nuclear damage and apoptosis changes in comparison with untreated cells. As projected, Ht-EAF fraction does not cause any adverse effects or apoptosis inducing effects to IR-HepG2. It was confirmed by DAPI, propidium iodide, rhodamine 123, and dual staining method (Figure 3b). Similar findings by Huang et al. (2015) confirmed that the phenolic-based phytoconstituents improved the glucose uptake in HepG2 cells [39]. Besides, the combined formulation of sun root and a fermented soybean (chungkookjang) ameliorated IR and improved the insulin signaling pathway in diabetic rats via regulating the gluconeogenesis [40]. These results clearly indicate that, the ethyl acetate fractions of Ht might have the potential to trigger the glucose uptake in IR-HepG2 cells.

### 3.6. Wound-Healing Ability in Fibroblast Cells

**Figure 4:**
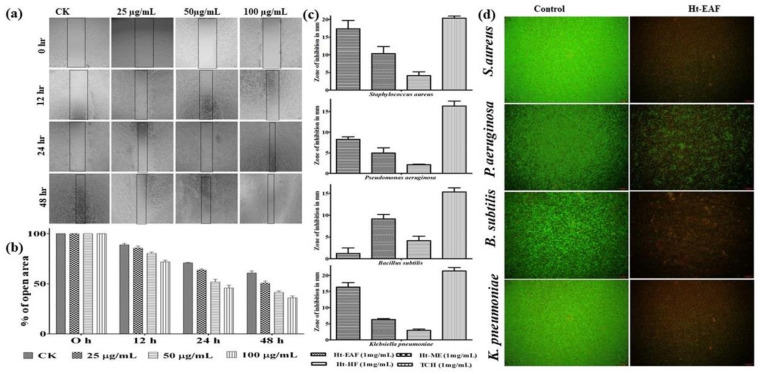
(**a**,**b**) Cell migration activity of different concentrations of Ht-EAF-treated NIH3T3 cells. (**c**) Zone of inhibition of Ht-EAF treated with different bacterial stains. (**d**) Live/Dead^TM^ BacLight^TM^ bacterial viability assay. Data were expressed as mean ± standard deviation of three independent experiments.

Foot ulcer is one of the threatening complications of DM, which could be serious if left untreated. About 14–25% of diabetic patients have the complication of foot ulcer. The fibroblast cells-based wound scratch assay is one of the inexpensive and well-recognized techniques to understand the wound-healing efficiency of natural products [41]. There are plenty of plant extracts playing an astonishing role in accelerating the healing process. But the possible mechanism behind their mode of action is still not understood properly. With this information, the cell migration assay was performed in fibroblasts of NIH 3T3 cells. Herein, the cells were treated with different concentrations (25, 50, 100 µg/mL) of Ht-EAF with an incubation period of 48 h. The cell-migration ability was captured for initial (0 h), mid (12 h and 24 h), and end of experimental period (48 h) and the wound closure distance was calculated using ImageJ software (Figure 4a,b). The untreated control cells showed a natural rate of cell migration, whereas the Ht-EAF showed a time-dependent manner of cell migration activity in NIH 3T3 cells. In all the cases, the natural rate of cell migration was notably lower than that of the tested concentration. A notable wound closure activity was noted for 100 µg/mL concentration of the extract.

The elevated levels of glucose in diabetic patients cause physiological dysfunction leading to delayed wound-healing process. The major factors affecting the wound-healing mechanism are colonization of aerobic bacterial species and generation of excessive amount of ROS, which extend the inflammatory phase [42]. The bacterial species such as *Staphylococcus aureus, Pseudomonas aeruginosa, Staphylococcus mutans, Bacillus subtilis, Klebsiella pneumoniae*, and *Escherichia coli* are commonly associated with diabetic foot ulcer [43]. The zone of inhibition studies and Live/Dead^TM^ BacLight^TM^ bacterial viability assay were performed to strengthen the findings of the wound-healing assay (Figure 4c). The results were compared with standard antibiotic of tetracycline and all tested solvent fractions of *H. tuberosus* exhibited marked antibacterial activity. The zone of inhibition was measured in the range of 1.24 to 17.35 mm. Among the tested fractions, ethyl acetate showed higher activity against *Staphylococcus aureus* (17.35 ± 1.34) followed by *Klebsiella pneumoniae* (16.34 ± 1.09), *Bacillus subtilis* (12.48 ± 1.27), and *Pseudomonas aeruginosa* (8.26 ± 0.61). These findings have also been confirmed by Live/Dead^TM^ BacLight^TM^ bacterial viability assay. Similar trend of results was also observed in case of ethyl acetate fraction treatment (Figure 4d). The above findings confirmed that ethyl acetate fraction of *H. tuberosus* shows a significant antibacterial activity. This led us to know that phenolic content in the ethyl acetate fraction is responsible for its antibacterial activity. There are several literatures evidences for the anti-bacterial activity of the flavonoid-rich fraction of plants [44,45]. Taken together, our results suggest that the identified phytocompounds might reduce the blood glucose level significantly and therefore, the wound-healing mechanism may proceed to next phase.

### 3.7. UPLC-QTOF-MS/MS Analysis

**Figure 5:**
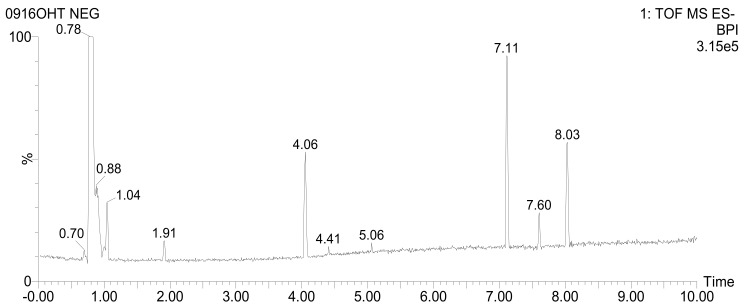
UPLC-QTOF-MS chromatogram of putatively identified compounds from ethyl acetate fraction of *H. tuberosus***.** RT at 1.04 min: fructosylnystose, 1.91 min: chlorogenic acid, 4.06 min: dicaffeoylquinic acid isomer1, 4.41 min: dicaffeoylquinic acid isomer2, 7.11 and 7.60 min: unknown, 8.03 min: pinellic acid.

As per the previously mentioned results, Ht-EAF exhibited the strongest radical scavenging ability and fascinating anti-diabetic effects. It might be due to the massive accumulation of bioactive compounds. Hence, Ht-EAF was utilized for the UPLC-QTOF-MS/MS analysis, and the findings are presented with tentatively identified phytocompounds along with formula, RT (min), *m*/*z* [M-H]^−^, mass error (ppm), response and fragmentation (*m*/*z*) (Figure 5 and Table 2).

The identified phytocompounds are in the nature of carbohydrates, amino acid, polyphenol, and their derivatives and few unknown compounds. From Ht-EAF, 21 compounds were identified by UPLC-QTOF-MS/MS analysis including phenolic compounds (neochlorogenic acid, chlorogenic acid, caffeic acid, and cryptochlorogenic acid), quinic derivatives compound (feruloylquinic acid, dicaffeoylquinic acids, and unsaturated carboxylic acid of cinnamic acid). First, sucrose was identified at the RT value of 0.79 min with deprotonated peak observed at *m*/*z* 341.1095 with MS/MS fragmentation at *m*/*z* 179.0563, followed by insulin-related oligosaccharides of fructosylnystose at 1.04 min, which exhibited a deprotonated peak at *m*/*z* 827.2669 with fragmentations at *m*/*z* 179.0560, 341.1090, 503.1615, 665.2151.

Chlorogenic acid belongs to the family of esters formed by the reaction between qunic acid and caffeic acid. [M−H]^−^ ions of three chlorogenic isomers, namely neochlorogenic acid, chlorogenic acid and cryptochlorogenic acid at RT of 1.50, 1.91 and 4.06 min, respectively, were detected at *m*/*z* 353.08 together with fragmentations at *m*/*z* 135.04, 179.03 and 191.05. These MS/MS fragmentations correspond to quinic and caffeic acids. Caffeic acid was also identified in RT at 2.37 min with *m*/*z* 179.0347. In addition, the [M−H]^−^ ions of feruloylquinic acid were observed at *m*/*z* 367.1028 with MS/MS fragmentation at *m*/*z* 134.0378, 173.0455, 191.0558, 193.0506. These results are in conformity with the previous findings of Chen et al. (2014a), who reported isolation of chlorogenic acid, dicaffeoylquinic acid, and feruloylquinic acid from the ethanolic extracts of Ht [46]. The phenolic glycoside of salicylic acid β-D-glucoside was eluted at 1.07 min and presented a deprotonated ion peak at 299.0772 with a molecular formula of C_13_H_16_O_8_. The phenolic glycoside derivatives including salvianolic acid derivative isomers 1, 2, and 3 at 3.91, 4.06, and 4.41 min, respectively, exhibited their corresponding deprotonated peaks at *m*/*z* 537.10 with MS/MS at *m*/*z* 135.04, 161.02, 179.03, 201.01, 375.06. The [M−H]^−^ ions and MS/MS fragmentations of the putatively identified compounds were compared with previously reported literatures (Table 2).

### 3.8. GC-MS Analysis

**Figure 6:**
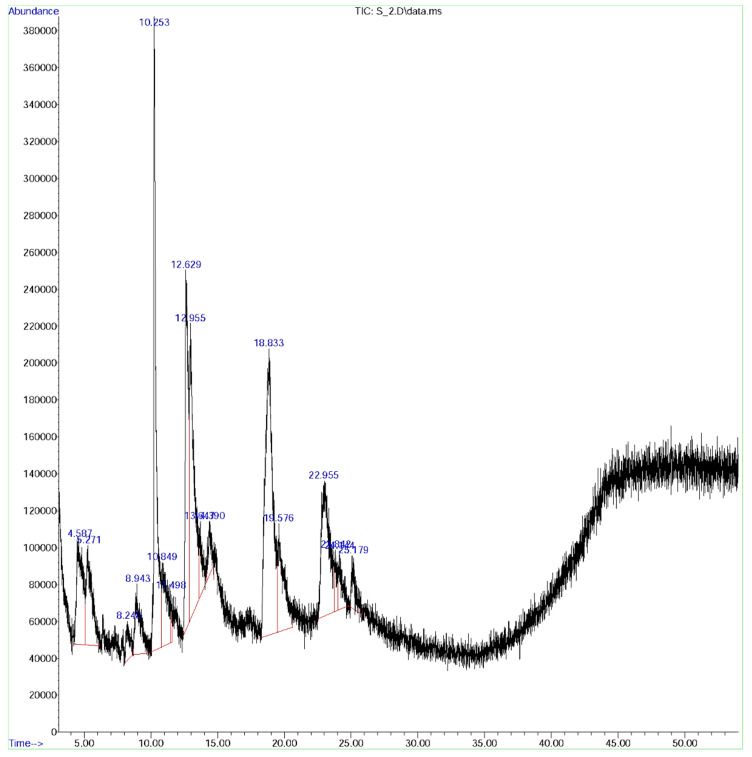
GC-MS chromatogram ofethyl acetate fraction of *H. tuberosus.*

Further, GC-MS analysis was employed to explore the phytochemical constituents of the ethyl acetate fraction of *H. tuberosus* (Figure 6). The GC-MS spectrum of the Ht-EAF was compared with the standard phytochemical library of W8N05ST.L and the name of the phytochemical constituents, RT value, area, structure, and pharmacological activities are summarized in Table 3. The first compound, caffeoylquinic acid was identified with less retention time of 4.58 min and the last compound, cyclopentanol was identified with the longest retention time of 25.182 min. The identified compounds possess diverse pharmacological activities. Caffeoylquinic acid has anti-bacterial, antioxidant, cardio protection, hepatoprotection, anti-pyretic, anti-inflammatory, anti-obesity, neuroptotection, anti-hypertension, anti-viral, and free radical scavenging activities [53]. Isoxazolidine (RT: 5.271; % of area: 4.47) enhances the hypoglycemic and hypolipidemic properties as well as controls the osmatic pressure in drug delivery system. Among the prevailing phytocompounds, β-bourbonene (RT: 8.921; % of area: 10.87) present in diverse plant families, has the ability to lower the blood glucose and lipid levels, and suppresses the oxidative stress in diabetic rats [54]. The phytocompounds, carbamic acid (RT: 10.253; % of area: 4.13), pent-4-enamide (RT: 12.629; % of area: 11.96), hexanoic acid (RT: 22.957; % of area: 10.20), and α-murrolene (RT: 23.011; % of area: 13.48) display significant and wide-ranging anti-microbial activity. Besides, the quinic acid derivative, feruloylquinic acid (R.t: 10.849; % of area: 13.77) possesses anti-cancer and anti-AIV (H5N1) activity [48]. 1,4-Dicaffeoylquinic acid (R.t: 18.833; % of area: 11.91), due to its phenolic nature, is believed to be a defense molecule against certain oxidative stress-related diseases [49]. The % of total area of phenolic nature of the phytocompounds were found to be 49.10 in GC-MS spectrum of the Ht-EAF. i.e., caffeoylquinic acid (5.37), isoxazolidine (4.47), feruloylquinic acid (13.77), 1,4-dicaffeoylquinic acid (19.91), and camphene (5.85). This finding is interconnected with the total phenolic content of ethyl acetate fractions, which reflects high amount of phenolic content. It was worth mentioning that the phenolic nature of the phytocompounds is responsible for the significant analgesic, anti-inflammatory, anti-oxidant, anti-microbial, anti-cancer, and anti-diabetic activity.

### 3.9. In Silico Conformation of Anti-Diabetic Efficacy

**Figure 7:**
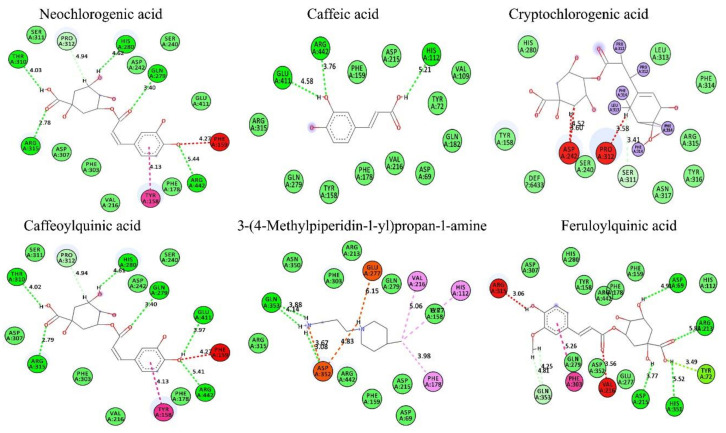
In silico docking analysis of isolated compounds from Ht-EAE with anti-diabetic target molecule of α-glucosidase.

**Figure 8:**
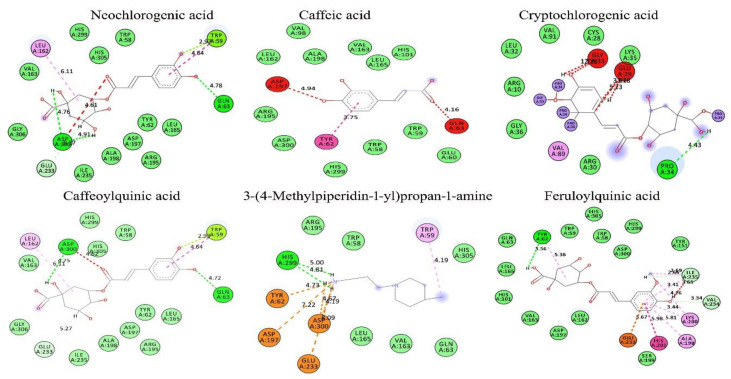
In silico docking analysis of isolated compounds from Ht-EAE with anti-diabetic target molecule of α-amylase.

Based on the findings of the GC-MS and antioxidant profile of the *H. tuberosus*, the in-silico docking studies were also performed by ArgusLab 4.01 and the results were interpreted using BIOVIA discovery studio visualizer V20. Based on the Lipinski’s hypothesis [71], the following phytocompounds of neochlorogenic acid, caffeic acid, cryptochlorogenic acid, caffeoylquinic acid, 3-(4-methylpiperidin-1-yl)propan-1-amine, and feruloylquinic acid were selected as ligands (Supplementary Table S1) and their molecular interactions were studied against anti-diabetic target of α-amylase and α-glycosidase (binding molecule) using molecular docking (Figure 7, Figure 8 and Supplementary Table S2)The docking analysis was performed by calculating the binding free energy (δG) based on varying electrostatic, C-H bond, hydrogen bond, and hydrophobic interactions and van der Wall effects. All the docking scores lie within −6.99116 to −10.7399 kcal/mol (i.e., low energy value). The binding energies of neochlorogenic acid, caffeic acid, cryptochlorogenic acid, caffeoylquinic acid, 3-(4-methylpiperidin-1-yl)propan-1-amine, and feruloylquinic acid against were −7.45264, −6.99116, −9.86082, −7.09424, and −8.61385 kcal/mol, respectively, with α-amylase and −10.7322, −8.28569, −10.632, −10.7379, −7.34669, and −9.31426 kcal/mol, respectively, with α-glycosidase.

Based on the hydrogen bonding ability and docking score, it can be concluded that cryptochlorogenic acid showed a superior binding affinity toward the binding molecule of α-amylase and caffeoylquinic acid against α-glycosidase. In the α-amylase docking assay, the docking score of cryptochlorogenic acid was found to be −9.86082 and it forms hydrogen bonds with amino acids, such as Glu318, Gly283, and Pro34, present in the active site, at a distance of 5.23 Å, 4.22 Å and 4.43 Å, respectively. Similarly, caffeoylquinic acid showed the highest binding score (−10.7379 kcal/mol) with α-glycosidase. It was found to form hydrogen bonds with amino acids of Arg442, Glu411, Gln279, His280, Thr310, and Arg315, present in the active site, at a distance of 5.41 Å, 3.97 Å, 3.40 Å, 4.61 Å, 4.02 Å, and 2.79 Å, respectively. Other tested compounds also have a significant docking ability.

## 4. Conclusions

This study explored the QTOF-MS- and GC-MS-based phytochemical analysis of *H. tuberosus* extracts and documented in vitro antioxidant, anti-diabetic, and wound-healing properties. The preliminary phytochemical studies revealed that the extract was enriched with phenolic and flavonoid compounds, which are responsible for their antioxidant activity. In addition, α-amylase and α-glycosidase inhibition assays were performed to document the anti-diabetic index of the *H. tuberosus* methanolic extract and its active fractions. In vitro findings showed that ethyl acetate fraction of the *H. tuberosus* did not show any remarkable cytotoxic features and there was no damage to the cell integrity tested with NIH3T3 cells. Besides, the ethyl acetate fraction has been shown to trigger the glucose uptake in IR-HepG2 and thereby reduce the diabetic condition. Our results suggest that the identified phytocompounds could reduce diabetes-related complications. As a future study, individual antidiabetic phytocompounds will be isolated and their anti-diabetic effects will be explored.

## Figures and Tables

**Table 1:** Total phenolic and total flavonoid content in different fractions and methanolic extract of *H. tuberosus*. GAE: Gallic acid equivalent; QE: quercetin equivalent.

	Total Phenolic Content (mg GAE/g of Extract)	Total Flavonoid Content (mg QE/g of Extract)
Ht-EAF	69.55 ± 0.36	21.03 ± 0.97
Ht-HF	17.11 ± 1.05	11.48 ± 0.11
Ht-MF	10.29 ± 0.82	6.27 ± 1.09
Ht-ME	43.07 ± 0.05	19.85 ± 0.21

**Table 2:** UPLC-QTOF-based mass spectra showing the fragmentation patterns of *H. tuberosus*.

RT (min)	Tentative Identification	Formula	*m*/*z* [M-H]-	Mass Error (ppm)	Response	Fragmentation (*m*/*z*)	Reference
0.79	Sucrose	C_12_H_22_O_11_	341.1095	1.9	3592134	179.0563	[47]
1.04	Fructosylnystose	C_30_H_52_O_26_	827.2669	−0.3	2910	179.0560, 341.1090, 503.1615, 665.2151	[47]
1.07	Salicylic acid β-D-glucoside	C_13_H_16_O_8_	299.0772	−0.1	315	137.0246, 180.0653	[48]
1.30	Phenylalanine	C_9_H_11_NO_2_	164.0717	−0.3	15377	147.0454	[49]
1.30	Cinnamic acid	C_9_H_8_O_2_	147.0457	3.5	2185	-	[50]
1.50	Neochlorogenic acid	C_16_H_18_O_9_	353.0879	0.2	4678	179.0345, 191.0557	[48]
1.75	Tryptophan	C_11_H_12_N_2_O_2_	203.0823	−1.5	4696	116.0516	[51]
1.91	Chlorogenic acid	C_16_H_18_O_9_	353.0876	−0.7	45770	135.0454, 179.0348, 191.0557	[48]
2.37	Caffeic acid	C_9_H_8_O_4_	179.0347	−0.3	2330	135.0457	[50]
2.54	4-*O*-(4-Coumaroyl)quinic acid	C_16_H_18_O_8_	337.0920	−2.6	187	191.0561	[48]
2.86	Feruloylquinic acid	C_17_H_20_O_9_	367.1028	−1.9	5135	134.0378, 173.0455, 191.0558, 193.0506	[48]
3.91	Dicaffeoylquinic acid isomer 1	C_25_H_24_O_12_	515.1196	0.3	9330	135.0460, 173.0455, 179.0349, 191.0557, 353.0873	[48]
3.91	Salvianolic acid derivative isomer 1	C_27_H_22_O_12_	537.1017	−4.1	1294	135.0457, 161.0245, 201.0171, 375.0706	[49]
4.06	Dicaffeoylquinic acid isomer 2	C_25_H_24_O_12_	515.1196	0.2	94316	135.0457, 173.0456, 179.0348, 191.0560, 353.0878	[49]
4.06	Cryptochlorogenic Acid	C_16_H_18_O_9_	353.0878	−0.1	11708	135.0457, 173.0456, 179.0348, 191.0560	[49]
4.06	Salvianolic acid derivative isomer 2	C_27_H_22_O_12_	537.1016	−4.1	19809	135.0457, 161.0245, 179.0348, 201.0168, 375.0698	[49]
4.41	Dicaffeoylquinic acid isomer 3	C_25_H_24_O_12_	515.1188	−1.3	25919	135.0457, 173.0455, 179.0347, 191.0560, 353.0879	[48]
4.41	Salvianolic acid derivative isomer 3	C_27_H_22_O_12_	537.1014	−4.6	3150	135.0457, 161.0245, 179.0347, 201.0172, 375.0695	[49]
7.11	Unknown	C_41_H_56_O_7_	659.3939	−2.1	207461	599.3732	-
7.60	Unknown	C_13_H_25_NO_3_	242.1760	−0.5	83368	224.1656	-
8.03	Pinellic acid	C_18_H_34_O_5_	329.2335	0.4	167137	171.1028, 211.1336, 229.1440	[52]

**Table 3:** Structure of some major compounds identify in GC/MS analysis and their biological activity of ethyl acetate fractions of *H. tuberosus*.

Peak	Rt Value	Area %	Name of Constituent	Formula	Pharmacological Activity
1.	4.58	5.37	Caffeoylquinic acid	C_16_H_18_O_9_	Activator of NF-κB signaling and macrophage infiltration [55]
2.	5.271	4.47	Isoxazolidine	C_3_H_7_NO	Anti-cancer [56,57], anti-diabetic [58], anti-fungal hypolipidemic activity [59]
3.	8.921	10.87	ß-Bourbonene	C_15_H_24_	Anti-diabetic and insulinotropic [54]
4.	8.943	3.33	3-(4-Methyl piperidin-1-yl)propan-1-amine	C_9_H_20_N_2_	Anti-Alzheimer’s agent [60]
5.	10.849	13.77	Feruloylquinic acid	C_17_H_20_O_9_	Anti-diabetic cardiovascular disease [61]
6.	10.253	4.13	Carbamic acid	CH_3_NO_2_	Anti-microbial, antifibrinolytics [62]
7.	11.489	0.39	4-Amino-1,2,5-oxadiazol-3-ol	C_2_H_3_N_3_O_2_	Akt kinase inhibitor [63]
8.	12.629	11.96	Pent-4-enamide	C_5_H_9_NO	Anti-bacterial agent [64]
9.	13.645	1.98	Morpholinoacetonitrile	C_6_H_10_N_2_O	anti-inflammatory, anti-cancer [65]
10.	14.378	2.52	3-Pyrrolidinol	C_4_H_9_NO	Antioxidant [66]
11.	18.833	19.91	1,4 Dicaffeoyl quinic acid	C_25_H_24_O_12_	Antioxidant, anti-ulcer [67]
12.	19.577	5.85	Camphene	C_10_H_16_	Anti-hyperlipidemic and anti-inflammatory [68]
13.	22.957	10.20	Hexanoic acid	C_6_H_12_O_2_	Anti-microbial [39]
14.	23.011	13.48	α-Murrolene	C_15_H_24_	Analgesic, anti-inflammatory, anti-bacterial, and anti-fungal activity [69]
15.	25.182	1.66	Cyclopentanol	C_5_H_10_O	Oxidative stabilizer, anti-oxidants [70]

## Data Availability

The data presented in this study are available on request.

## Supplementary Materials

The following are available online at https://www.mdpi.com/2076-3921/10/1/99/s1. Table S1: Lipinski’s rule for of GC-MS-based isolated compound from *H. tuberosus* assessed by the web tool of SwissADME. Table S2: Molecular interaction of the α-amylase and α-glycosidase active site with selective phytocompound inhibitors from *H. tuberosus*.
