# Supplementary material for: Ethyl Acetate Fraction of Helianthus tuberosus L. Induces Anti-Diabetic, and Wound-Healing Activities in Insulin-Resistant Human Liver Cancer and Mouse Fibroblast Cells
Source: Antioxidants (Basel). 2021 Jan 12;10(1):99. doi: 10.3390/antiox10010099 (PMC7828129; doi:10.3390/antiox10010099)
Supplement: Supplementary file 1 [file antioxidants-10-00099-s001.pdf]

Supplementary Table 1. Lipinski's rule for of GC-MS based isolated compound from *H.tuberosus* assessed by the web tool of SwissADME.

Compound	Molecular Weight (Da)	Hydrogen Bond		LogP	Molar Refractivity	Rules Satisfied
		Donor	Acceptor			
Salicylic acid β-D-glucoside	299.07	5	8	0.98	67.55	5/5
Neochlorogenic acid	353.08	6	9	0.96	83.50	4/5
Caffeic acid	179.03	3	4	0.97	47.16	5/5
5-O-(4-Coumaroyl)-D-quinic acid	337.09	5	8	1.08	81.48	5/5
Feruloylquinic acid	367.10	5	9	1.47	87.97	5/5
Cryptochlorogenic acid	353.08	6	9	1.23	83.50	5/5
Pinellic acid	329.23	4	5	2.24	93.42	5/5
Caffeoylquinic acid	354.31	6	9	0.96	83.50	4/5
Isoxazolidine	73.09	1	2	1.22	22.22	4/5
ß-Bourbonene	204.35	0	0	3.34	67.14	3/5
3-(4-Methylpiperidin-1-yl)propan-1-amine	156.27	1	2	2.29	52.78	5/5
Carbamic acid	61.04	2	2	0.20	11.79	4/5
4-Amino-1,2,5-oxadiazol-3-ol	101.06	2	3	0.20	21.53	4/5
Pent-4-enamide	99.13	1	1	1.20	28.58	4/5
Morpholinoacetonitrile	126.16	0	3	1.49	36.49	3/5
3-Pyrrolidinol	87.12	2	2	1.20	27.11	4/5
1,4 Dicaffeoylquinic acid	516.45	7	12	1.91	126.60	3/5
Camphene	136.23	0	0	2.58	45.22	3/5
Hexanoic acid	116.16	2	4	1.57	32.73	4/5
α-murrolene	204.35	0	0	3.38	69.04	3/5
Cyclopentanol	86.13	1	1	1.59	25.20	4/5

Supplementary Table 2. Molecular interaction of the α-Amylase and α-Glycosidase active site with selective phytocompound inhibito from H.tuberosus.

PubChem ID	Phytocompound	Docking Score	No of H-bond	Interactive residues	
				H-Bond	Hydrophobic bond
			**α-Amylase**		
5280633	Neochlorogenic acid	-7.45264	2	Asp300, Gln63, Glu233	TRP59,LEU162,Asp300,
689043	Caffeic acid	-6.99116	3	Arg389, Glu390, Cys378	Arg389,Trp388, Cys384, Thr377
9798666	Cryptochlorogenic acid	-9.86082	4	Glu390, Glu484, Val383, Arg343	Glu484, Ala318,Val348
10155076	Caffeoylquinic acid	-9.32055	3	Asp300, Glu233, Gln63	TRP59, Asp300, Leu162
3149264	3-(4-Methylpiperidin-1-yl)propan-1-amine	-7.09424	2	His299	Trp59, Tyr62, Asp197, Glu233, Asp300
10133609	Feruloylquinic acid	-8.61385	3	Tyr62, Ile235, Val234	Tyr62, Ile235, Lys200, Ala198, His201, Glu233
			**α-Glycosidase**		
5280633	Neochlorogenic acid	-10.7322	5	Arg442,Gln279, His280, Thr310, Arg315	Pro312, Phe159, Tyr158
689043	Caffeic acid	-8.83794	3	Phe433,Ser236,Lys156	Ile419,Lys156
9798666	Cryptochlorogenic acid	-10.632	4	Ser236,Asn317,His423,Asn235	Asn415,Phe314,His423
10155076	Caffeoylquinic acid	-10.7379	6	Arg442,Glu411,Gln279, His280,Thr310,Arg315	Pro312, Phe159,Tyr158
3149264	3-(4-Methylpiperidin-1-yl)propan-1-amine	-7.34669	3	Asp352,Gln353	Asp352,Glu377,Val216,Tyr158, His112, Phe178
10133609	Feruloylquinic acid	-9.31426	4	Asp69, Arg213, His351, Asp215	Tyr72, Gln279, Phe303, Gln353, Arg315
